# Electrophysiological Markers of Aberrant Cue-Specific Exploration in Hazardous Drinkers

**DOI:** 10.5334/cpsy.96

**Published:** 2023-07-28

**Authors:** Ethan M. Campbell, Garima Singh, Eric D. Claus, Katie Witkiewitz, Vincent D. Costa, Jeremy Hogeveen, James F. Cavanagh

**Affiliations:** 1 Department of Psychology & Psychology Clinical Neuroscience Center, University of New Mexico, US; 2 Department of Biobehavioral Health, Pennsylvania State University, US; 3 Division of Neuroscience, Oregon National Primate Research Center, US

**Keywords:** explore-exploit, addiction, P3a, alcohol, POMDP

## Abstract

**Background:**

Hazardous drinking is associated with maladaptive alcohol-related decision-making. Existing studies have often focused on how participants learn to exploit familiar cues based on prior reinforcement, but little is known about the mechanisms that drive hazardous drinkers to explore novel alcohol cues when their value is not known.

**Methods:**

We investigated exploration of novel alcohol and non-alcohol cues in hazardous drinkers (N = 27) and control participants (N = 26) during electroencephalography (EEG). A normative computational model with two free parameters was fit to estimate participants’ weighting of the future value of exploration and immediate value of exploitation.

**Results:**

Hazardous drinkers demonstrated increased exploration of novel alcohol cues, and conversely, increased probability of exploiting familiar alternatives instead of exploring novel non-alcohol cues. The motivation to explore novel alcohol stimuli in hazardous drinkers was driven by an elevated relative future valuation of uncertain alcohol cues. P3a predicted more exploratory decision policies driven by an enhanced relative future valuation of novel alcohol cues. P3b did not predict choice behavior, but computational parameter estimates suggested that hazardous drinkers with enhanced P3b to alcohol cues were likely to learn to exploit their immediate expected value.

**Conclusions:**

Hazardous drinkers did not display atypical choice behavior, different P3a/P3b amplitudes, or computational estimates to novel *non-alcohol* cues—diverging from previous studies in addiction showing atypical generalized explore-exploit decisions with non-drug-related cues. These findings reveal that cue-specific neural computations may drive aberrant alcohol-related decision-making in hazardous drinkers—highlighting the importance of drug-relevant cues in studies of decision-making in addiction.

## Introduction

Hazardous alcohol consumption is a major risk factor for development of alcohol use disorder (AUD) (Kranzler & Soyka, 2018) and is characterized by measurable deficits in adaptive decision-making (Kovács et al., 2017) and associated functional brain activity (Galandra et al., 2018). Alcohol cues elicit elevated mesocorticolimbic activity among individuals who engage in hazardous drinking (Filbey et al., 2008) and alcohol cue reactivity is tied to drinking outcomes in clinical trials for AUD (Miranda et al., 2020), yet a satisfactory neurobehavioral framework for maladaptive alcohol-related decision-making is lacking (Bickel et al., 2018). This gap in the literature may impede tailoring treatment to address substance-related health disparities (Sprague Martinez et al., 2018), especially given neural changes tied to childhood neglect and early alcohol use (Nooner et al., 2022). One promising yet understudied area of decision-making in addiction is the explore-exploit tradeoff—the motivational tension between exploring novel choice options versus sticking with familiar options to maximize short-term rewards (Addicott et al., 2017; Wilson et al., 2021). Heightened motivation to explore novel options likely plays a key role in the development and maintenance of hazardous drinking (Aloi et al., 2021; Qi et al., 2021), yet existing research has almost exclusively focused on reactivity to familiar alcohol cues (Zeng et al., 2021). Here, by merging explore-exploit decision-making in response to substance-related cues and concomitant neural recordings, we aimed to identify cognitive biomarkers of aberrant alcohol-specific exploration in hazardous drinkers.

In addition to assaying explore-exploit decision-making and neural responses to novel alcohol cues in hazardous drinkers, the current study also used computational modeling to formalize relationships between these levels of analysis. Computational psychiatry leverages mathematical models of normative behavior to better understand the mechanisms driving aberrant behaviors in patients with mental health challenges (Friston et al., 2017; Huys et al., 2016). In the context of explore-exploit decisions, an optimal behavioral strategy would be to make decisions that, over the long-term, maximize gains or minimize losses by predicting *both* the immediate and anticipated future consequences of each available choice option (Averbeck, 2015; Wilson et al., 2014). Thus, the decision to exploit is motivated by the anticipated probability that a choice will be rewarded immediately based on prior reinforcement, whereas prospection about how often a choice will likely be rewarded in the future drives the difficult decision to explore a novel option. While prior studies have often focused on the neural mechanisms of reinforcement in addiction (Koob, 1992; Lüscher et al., 2020), the current study is the first to use a model to formally estimate the relative increase in future value (Averbeck, 2015) to be gained by exploring novel alcohol versus non-alcohol cues. A better neurocomputational characterization of the future value signals that motivate hazardous drinkers to explore novel alcohol cues could contribute to refining existing methods of classifying and diagnosing AUD, predicting risky drinking behavior, and establishing more sensitive markers of treatment-related change.

Electroencephalography (EEG) is ideal for studying time-locked neural computations underlying novelty-directed behaviors, particularly the P3 family of event-related potential (ERP) components which are canonical EEG features 300–600 milliseconds (ms) after the presentation of a salient stimulus. The P3a, which has a mid-frontal topographical distribution, responds to novel stimuli and is thought to reflect a top-down novelty orienting response (Polich, 2007). The target-evoked P3b has a posterior parietal distribution and is thought to relate to context updating (Donchin & Coles, 1988) and accumulation of information leading to a decision (Cavanagh, 2015; Rac-Lubashevsky & Kessler, 2019; Twomey et al., 2015). The P3a and P3b are simple and precise EEG measures supported by decades of research, including in the study of AUD (Pfefferbaum et al., 1979). Both responses are found to be altered across the spectrum of alcohol use depending on task demands (Campanella et al., 2019) and whether or not alcohol cues are presented (Martins et al., 2022). Together, ERPs and computational models of behavior offer an ideal combination for addressing aberrant time-locked neural computations that may occur in clinically relevant populations.

We recorded EEG during a probabilistic reinforcement learning task commonly used to assay explore-exploit decision-making (Costa & Averbeck, 2020; Costa et al., 2019, 2014; Djamshidian et al., 2011; Hogeveen et al., 2022; Wittmann et al., 2008), which included periodic insertions of novel alcohol and non-alcohol beverage stimuli. We hypothesized that hazardous drinkers would demonstrate heightened P3a responses and greater future value-motivated exploratory decision-making in response to novel alcohol cues relative to age- and gender-matched control participants. Additionally, we hypothesized that P3b amplitudes would be higher to novel alcohol cues in hazardous drinkers, given their intrinsic motivational significance relative to non-alcohol cues. Overall, our behavioral, computational, and ERP results converged to suggest novel neurocomputational markers of alcohol-specific explore-exploit behavior in hazardous drinkers.

## Methods and Materials

### Inclusion and exclusion criteria

All participants were between the ages of 18 and 55, fluent in English with no history of seizure, no neurological impairment or learning disorder, no current use of illicit substances, no history of head trauma resulting in loss of consciousness for over 5 minutes, and were not seeking or receiving treatment for AUD. Hazardous drinking participants had an Alcohol Use Disorders Identification Test (AUDIT) (Bush et al., 1998; Saunders et al., 1993) score >7 for men and >6 for women. Control participants had to have an AUDIT score <4 prior to participation during a phone screening and at the time of the experiment. Hazardous drinkers were recruited from ABQDrinQ (R01AA023665), a separate study conducted by investigators from the UNM Center on Alcohol, Substance use, and Addictions (CASAA) and the Mind Research Network (MRN) to assess neurocognitive patterns associated with changes in the drinking behavior of non-treatment seeking individuals with AUD (Al-Khalil et al., 2021).

### Participants

30 participants (17 female) were recruited from ABQDrinQ for the hazardous drinking sample. These participants were invited back to the present study 18–32 months later and they were re-assessed with the AUDIT. 28 controls (16 female) were recruited from other studies and the community. Five participants were excluded after data collection (one control participant had a high AUDIT score on test day, two had poor EEG data quality, and two were outliers (*SD* ≥ 3) on our outcome measures). The final sample comprised *n* = 27 (15 female) in the hazardous drinking group and *n* = 26 (15 female) controls. Table S1 includes descriptive statistics for demographic and questionnaire data. There were no significant differences between groups for sex (*p* = 0.874), tobacco/nicotine use (*p* = 0.638), ethnicity (*p* = 0.933), race (*p* = 0.231), age (*p* = 0.741), or years of education (*p* = 0.151). The groups did significantly differ for AUDIT (*p <* .001) and Beck Depression Inventory (BDI) scores (Beck et al., 1988) (*p* = 0.023), with BDI differences being expected given the greater severity of depressive symptoms reported in hazardous drinkers (Lannoy et al., 2021). Additionally, within the hazardous drinking group, BDI and AUDIT scores were correlated (*rho*(25) = 0.445, *p* = 0.018).

### Procedure

**Figure 1:**
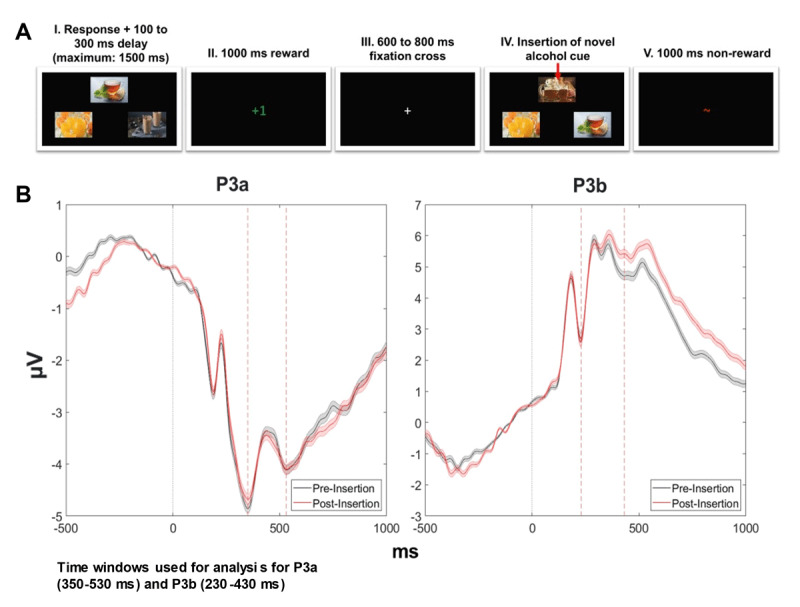
**(A)** Three-armed alcohol novelty bandit task schematic. Displays reward and non-reward presentations as well as the novel insertion of an alcohol cue. **(B)** P3a and P3b averaged across all participants and cue types with highlighted time windows of analysis prior to (pre-insertion) and one trial after novel insertions (post-insertion) with error bars +/– SEM.

Participants completed paper questionnaires including the AUDIT and BDI followed by a series of computerized tasks including the three-armed bandit task with alcohol and non-alcohol beverage stimuli (Figure 1a). The task consisted of 350 trials in which participants chose between three images that were probabilistically associated with a reward. Each trial comprised i) a jittered duration fixation cross (600–800 ms), ii) a choice event (3 targets presented in the upper, left, or right area pseudo-randomly assigned; participants had 1500 ms to select one of the images by pressing buttons corresponding to their locations), iii) a jittered duration blank screen (100–300 ms), and iv) a feedback event (+1 or ~ ; 1000 ms; Figure 1a). If the participant failed to respond in time, a null signal (“No Response Detected”) was displayed (≈2.97% of trials per participant). During the experiment, 50 images (split evenly between alcohol and non-alcohol stimuli) were introduced which randomly replaced one of the existing options with a minimum of 5 trials and a maximum of 9 trials between novel insertions (# trials between novel insertions: *M* = 6.86, *SD* = 1.63). At the start of the experiment, the three initial choices were randomly assigned a reward probability of 0.2, 0.5, or 0.8. Novel choice options were also randomly assigned one of these reward probabilities when introduced. No more than two of the three options could be assigned the same reward probability at a time. Participants were instructed to win as many points as possible by choosing the image that rewarded them most often. They were also told about the probabilistic nature of the rewards and that the images’ positions did not affect the probability of receiving a reward.

### Beverage Image Set and Participant Ratings

An image rating task was administered to obtain ratings of liking of various image categories. Participants were presented with an image from one of five categories: puppies, scenes of nature, babies, alcohol, neutral (e.g., a chair), and negative images (e.g., garbage). Pictures were selected from web image searches (e.g., “high-definition puppy images”). Six images from each category were presented (a total of 36 images). This task was previously reported (Brown et al., 2022), but here alcohol images substituted cow images. Participants rated how pleasant they felt the image was on a scale from 1 (very unpleasant) to 9 (very pleasant). Both controls and hazardous drinkers rated all classes of affective imagery similarly except for the alcohol images, which were rated more pleasant in hazardous drinkers (*p <* .001). Within hazardous drinkers, AUDIT score correlated significantly with pleasantness ratings for alcohol images (*rho*(25) = 0.34, *p* = 0.041). Therefore, the chosen image set demonstrates face validity for the salience of alcohol images to hazardous drinkers.

### Computational Modeling

Optimal explore-exploit decision-making was modeled based on estimates of state and action values derived from a partially observable Markov decision process (POMDP) (Averbeck, 2015). This allows estimation of the utility of choosing particular options (i.e., actions) at particular timepoints (i.e., states). The utility of each information state changes trial-by-trial and varies as a function of the action that maximizes total action value. The action values in each state are the sum of the immediate (IEV) and future (FEV) expected values of choosing a particular option. IEV is an estimate of the likelihood that a given option will be rewarded based on prior outcomes, while FEV reflects the discounted future gains or losses that can be expected given what is learned after choosing a particular option. The IEV is easy to compute, based on explicit positive or negative feedback about prior decisions. In contrast, the FEV is more difficult to compute and involves simulating the number of future gains or losses that might result after choosing a particular option. Notably, because subjects could choose freely among the three-arms in the bandit task, the FEV of a given option is highly tied to the overall richness of the current choice set. FEV is high if the best available option(s) presented have a high versus a low IEV. The state space and formalism of the POMDP has been described in depth elsewhere (Averbeck, 2015; Costa et al., 2019; Hogeveen et al., 2022), with the only difference here being that the weighted utility functions were computed separately for alcohol and non-alcohol stimuli.

The FEV of one option compared to the average FEV across all options is crucial to determining the relative increase or decrease in future value associated with exploration. We refer to this relative FEV quantity as the exploration BONUS. Immediately after a novel option is added to the choice set, uncertainty about its IEV is high, and the BONUS associated with choosing that option relative to familiar alternatives is at its highest. This BONUS value then decreases over trials as the subject samples the novel option and learns its IEV. In contrast, the BONUS values associated with familiar alternative options are negative when a novel option is introduced. This occurs because the subject has already sampled those options, lowering their FEV relative to the novel stimulus. Notably, as the subject forgoes these familiar alternatives to explore the novel stimulus, their FEV and BONUS values steadily increase over trials.

In sum, within this POMDP framework BONUS values above 0 are associated with exploratory choices (i.e., driven by small relative differences in FEV of a given option), whereas BONUS values below 0 are associated with exploitative choices. To be clear, we do not think participants mentally simulate all possible choice sequences to estimate exploration bonuses prior to each choice. But given the fact that the POMDP model-derived estimates fit well with observed choice behavior on the current task across nonhuman primates and human subjects (Costa & Averbeck, 2020; Costa et al., 2019; Hogeveen et al., 2022)—this modeling approach does capture meaningful variance in the way individuals utilize uncertainty about the value of new information to motivate the decision to explore novel choice options.

### EEG Data Preprocessing and Analysis

EEG was recorded on a 64-channel Brain Vision system (Brain Products GmbH, Munich, Germany) between .01–100 Hz at a sampling rate of 500 Hz. CPz was used as the reference electrode and FPz as the ground electrode. Electrocardiogram (EKG) was recorded as was vertical electrooculogram (VEOG) via two auxiliary electrodes placed above and below the left pupil. All EEG preprocessing was executed using custom Matlab scripts and EEGLAB (Delorme & Makeig, 2004) functions. Data were band-pass filtered between 0.1–20 Hz and independent components analysis (ICA) was used to remove eyeblinks. Data were average referenced and poor-quality electrodes and epochs were removed after visually inspecting data. EEG data were epoched at –500 to 1000 ms relative to stimulus presentation, baseline corrected, and then averaged to generate ERPs.

ERP regions of interest were selected from electrodes which were closest to maximal activation along the midline during and one trial after novel insertions. The P3a is elicited by novel distracting stimuli (Donchin & Coles, 1988; Pfefferbaum et al., 1979) and was expected to be enhanced due to novel insertions. The P3b is elicited by cues with enhanced motivational significance (Cavanagh, 2015; Nieuwenhuis et al., 2005) such as cues with higher reward probabilities. P3a was quantified at electrode FCz and P3b was quantified at electrode POz. These distinct ERP components were calculated by taking the average across time windows of peak activation between 350–530 ms for P3a and 230–430 ms for P3b (Figure 1b; Polich, 2007).

### Modeling Approach and Outlier Removal

To test hypotheses about the neural substrates of explore-exploit behavior in hazardous drinkers, data were modeled as a function of both (1) choices following novel options, and (2) computational parameters reflecting decision-making behavior. Specifically, individual differences in subjects’ explore-exploit decision-making tendencies based on latent value parameters, IEV and exploration BONUS, from the POMDP model associated with alcohol and non-alcohol choice options. Linear mixed effects models were computed with group and cue type as fixed effects and participant as a random effect. ERP trial outliers were removed when they exceeded a Mahalanobis’ distance of 13.82 (for 2 predictors at alpha = 0.001). Estimated marginal means with two test false discovery rate correction were used for paired comparisons of conditions between groups.

### Evaluating Assumptions of the General Linear Model

Across both the alcohol and non-alcohol novel stimulus conditions, our EEG components of interest (P3a, P3b) and key computational model-derived parameters of interest (BONUS, IEV) violated the assumption of normality (*p*s ≤ 0.023). Additionally, the probability of exploring a novel stimulus, EEG components of interest, and BONUS parameters all violated the assumption of homoscedastic variances across both groups (all *p*s ≤ 0.044). Due to these violations of the assumptions of the general linear model, we used robust variants of all inferential tests where stimulus condition or participant group were independent predictors (Field & Wilcox, 2017). Specifically, we computed robust linear mixed effects models that down-weighted observations with excessively high residual values to reduce their impact on model estimates (Koller, 2016). Discrete explore-exploit behaviors were measured via participant’s selection of the novel option (i.e., exploration), the best alternative (i.e., exploiting the familiar option with highest reward probability), or the worst alternative (i.e., familiar option with lowest reward probability) on the first trial after a novel insertion (Hogeveen et al., 2022). In addition to discrete explore-exploit behaviors on the early post novel insertion trial, subject-specific POMDP coefficients enabled us to model continuous variation in the value of exploration (i.e., BONUS) and exploitation (i.e., IEV) for alcohol or non-alcohol stimuli across all trials.

## Results

### Increased Exploration of Novel Alcohol Cues in Hazardous Drinkers

**Figure 2:**
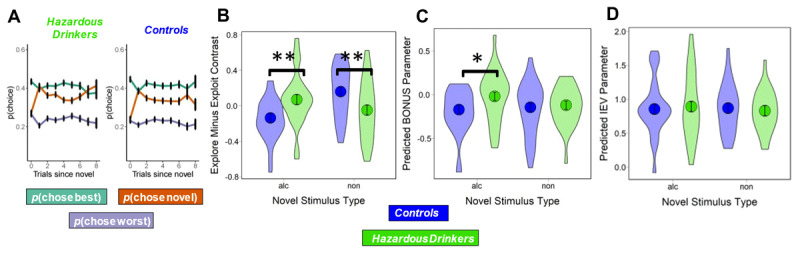
**(A)** Choice behavior averaged across both stimulus types between groups over trials since a novel insertion for the novel, best alternative, and worst alternative stimuli. Note the increase in exploratory behavior on trial 1 after a novel insertion. **(B)** Probability of selecting the novel stimulus (exploration) minus probability of selecting the best alternative (exploitation) on the post-insertion trial. Hazardous drinkers explored alcohol stimuli more often than controls, and controls explored non-alcohol stimuli more often than hazardous drinkers. **(C)** BONUS estimates with hazardous drinkers having higher BONUS values for alcohol cues than controls. **(D)** IEV estimates with no significant differences between groups or cue types.

Periodic novel stimulus insertions explicitly forced participants to make explore-exploit choices. Participants did not tend to explore on the insertion trial and instead waited until the next trial (post-insertion) to sample new stimuli (Figure 2a). To directly quantify explore-exploit behavior, we computed the choice probability of selecting the novel stimulus (exploration) versus the best available alternative (exploitation) on the first post-insertion trial. Participants tended to exploit more often than they explored (Figure 2a; *MD* = 0.063; t(52) = 2.73, *p* = 0.008), but in turn they also explored more often than they chose the worst stimulus (Figure 2a; *MD* = 0.064; t(52) = 3.78, *p <* .001).

To evaluate how participants responded to the presentation of each novel cue type, we subtracted exploit choice probability from explore choice probability. To test the effect of cue type on explore-exploit behavior between groups, we ran a model predicting choice behavior from group and cue type (b = –0.416, SE = 0.083, *p* < .001) and found that hazardous drinkers explored alcohol stimuli more often than controls (Figure 2b; b = –0.208, SE = 0.079, *p* = 0.009). Controls also explored non-alcohol stimuli more often than hazardous drinkers (Figure 2b; b = 0.208, SE = 0.079, *p* = 0.009), demonstrating alcohol cue-specific exploration in hazardous drinkers relative to controls.

### Increased Exploration BONUS for Alcohol Cues in Hazardous Drinkers

Within the POMDP framework, immediate expected value (IEV) is an estimate of the likelihood that a given option will be rewarded based on prior outcomes. When a novel option is introduced, the uncertainty about its IEV is high, and the exploration BONUS associated with choosing the novel option vs. familiar alternatives is at its highest. POMDP model-derived estimates for all choices correlated with choice behavior for each participant (*M* = 0.737, *SD* = 0.267, 95% CI = 0.6–0.84) and this correlation did not differ between controls and hazardous drinkers (t = 0.308, *p* = 0.758). Subject-level POMDP estimated coefficients reflected each individual’s degree of reliance on the exploration BONUS and IEV signals in shaping their decision-making separately for alcohol (*rho*(51) = –0.192, *p* = 0.167) and non-alcohol cues (*rho*(51) = –0.298, *p* = 0.031). To test whether POMDP parameters related to group and cue type we ran two models, one predicting BONUS and one predicting IEV from group and cue type. We found that BONUS values were predicted by a two-way interaction between group and cue type (b = –0.126, SE = 0.059, *p* = 0.038). Hazardous drinkers had higher BONUS values for alcohol cues than did controls (Figure 2c; b = –0.15, SE = 0.07, *p* = 0.031). No other main effects were significant, and IEV coefficients did not differ as a function of group, cue type, or their interaction (Figure 2d). Building off the post-insertion trial behavioral results, these differences in the BONUS parameter demonstrate that hazardous drinkers continued alcohol-specific exploration across trials.

### Increased P3a with Exploration of Alcohol Cues in Hazardous Drinkers

**Figure 3:**
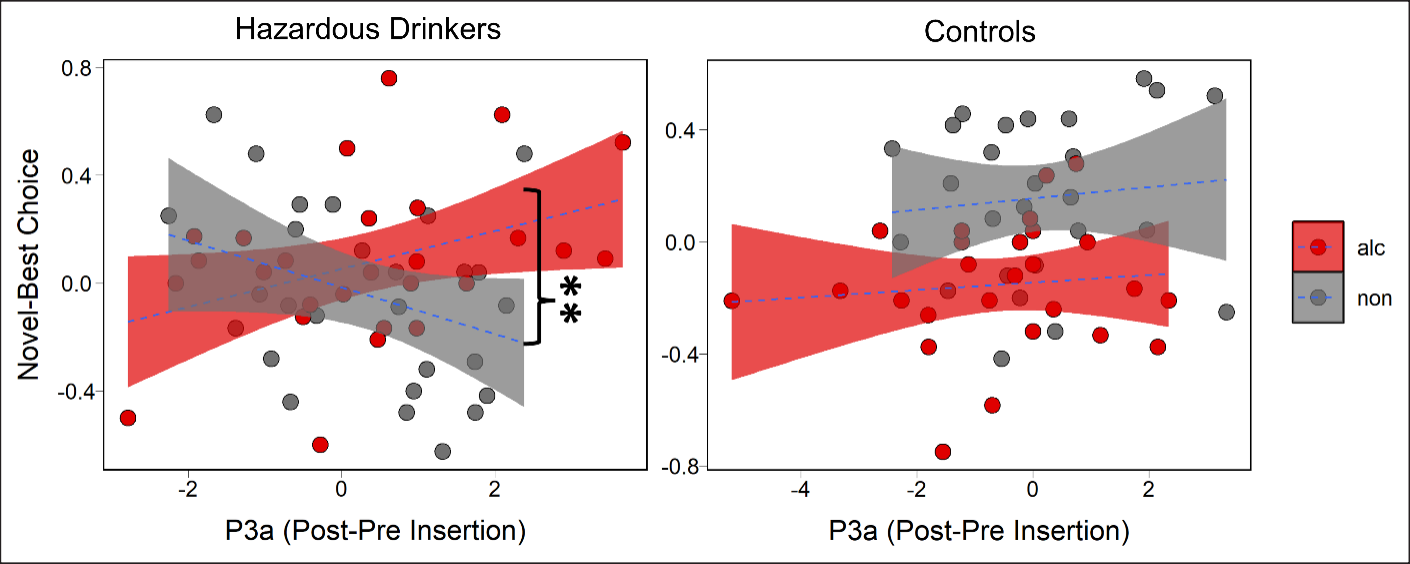
Probability of explore minus exploit behavior by P3a, contrasted by group and cue type with significant paired comparison marked. For hazardous drinkers but not controls, an increase in the P3a is associated with an increase in the exploration of alcohol cues relative to non-alcohol cues.

P3a and P3b amplitudes were calculated as the difference in the amplitude between the ERP elicited by the presentation of the three choice stimuli on the trial after a novel option was introduced (i.e. post-insertion trial) minus the ERP elicited by the immediately preceding trial (i.e. pre-insertion trial) (Figure 1b). Prior research indicates that novel minus non-novel difference waves can be useful for distinguishing clinical populations (Kappenman & Luck, 2016) and have been used to do so for assessing novelty-sensitive neural responses (Bertram et al., 2020). To test whether each P3 component related to cue-specific explore-exploit behavior between groups, we ran two models predicting choice behavior from group and cue type, one with P3a amplitude and one with P3b amplitude as additional predictors. We found that probability of selecting the novel stimulus was predicted by a three-way interaction between group, chosen cue type, and P3a (b = 0.155, SE = 0.068, *p* = 0.025). In hazardous drinkers, the decision to explore novel alcohol cues was associated with an increase in the P3a relative to controls (Figure 3; b = 0.133, SE = 0.05, *p* = 0.007). P3b amplitude was not influenced by explore-exploit tendencies toward alcohol or non-alcohol cues in controls or hazardous drinkers (b = –0.045, SE = 0.08, *p* = 0.575). In sum, hazardous drinkers were specifically characterized by a P3a marker of alcohol-specific exploratory behavior.

### Increased P3a with Exploration BONUS for Alcohol Cues in Hazardous Drinkers

**Figure 4:**
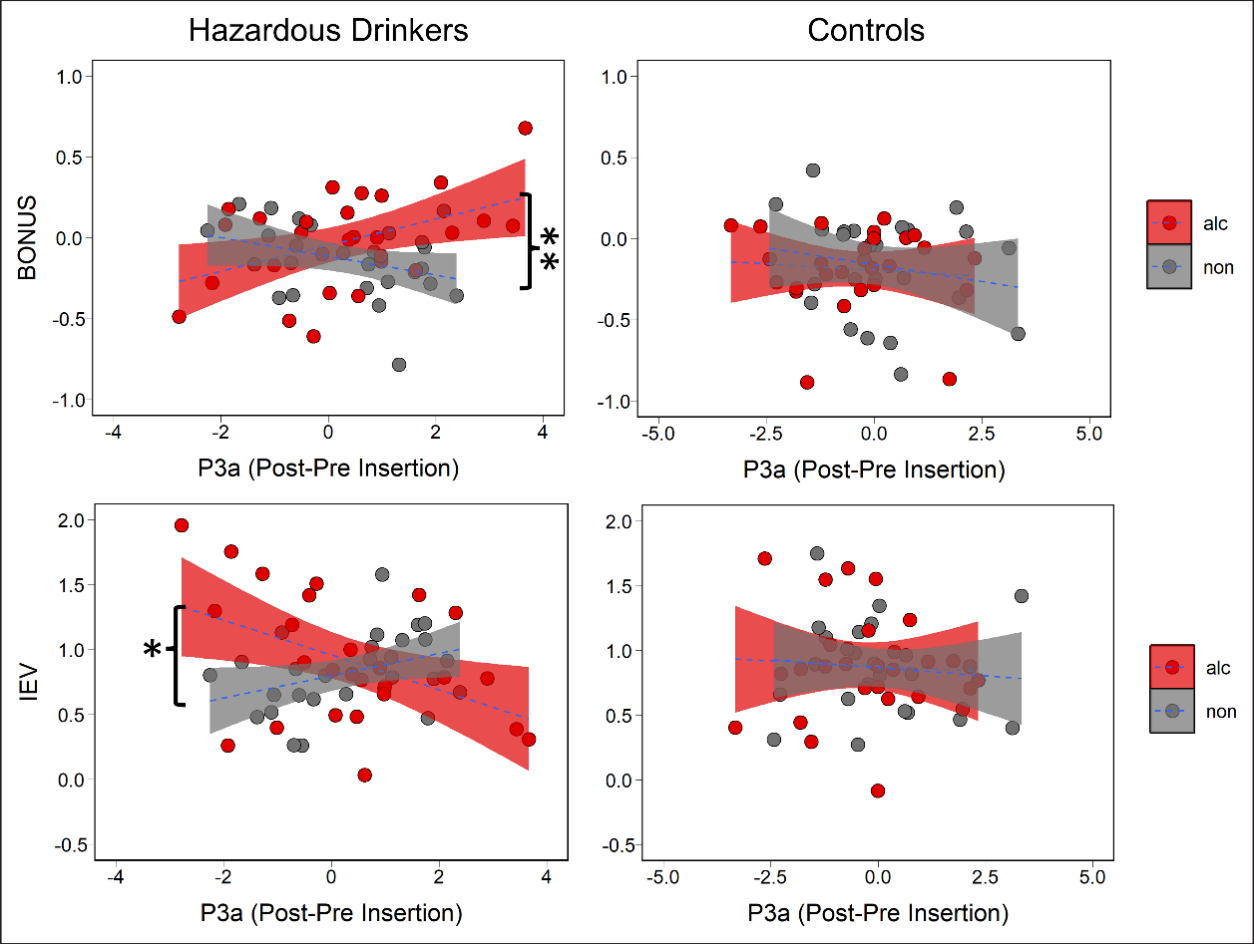
BONUS and IEV parameters by P3a amplitude split by group and cue type with significant paired comparisons marked. For hazardous drinkers but not controls, an increase in the P3a is associated with the BONUS values for alcohol cues relative to non-alcohol cues. Additionally in hazardous drinkers but not controls, the P3a decreases as the IEV of alcohol cues increases relative to non-alcohol cues.

**Figure 5:**
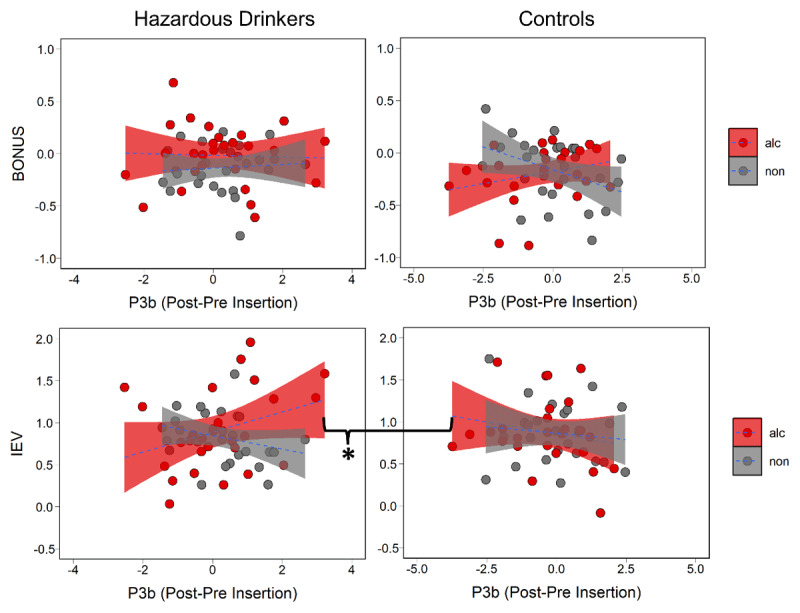
BONUS and IEV parameters by P3b amplitude split by group and cue type with significant paired comparison marked. The IEV of alcohol cues increases with P3b amplitude in hazardous drinkers relative to controls.

To test whether the P3a component related to cue-specific POMDP parameters between groups, we ran two models with group, cue type, and P3a amplitude as predictors for BONUS in one model and IEV in the other. We found that BONUS coefficients were predicted by a two-way interaction between chosen cue type and P3a amplitude (b = –0.116, SE = 0.039, *p* = 0.004). The BONUS and P3a had a stronger relationship for alcohol relative to non-alcohol stimuli across groups (b = 0.078, SE = 0.027, *p* = 0.004). Separated by group, this difference was only apparent in hazardous drinkers (Figure 4; b = 0.04, SE = 0.037, *p* = 0.003). This demonstrated that as with exploration, the P3a increases as the BONUS values for alcohol cues increase. IEV coefficients were predicted by a three-way interaction between group, chosen cue type, and P3a (b = –0.227, SE = 0.082, *p* = 0.007). Paired comparisons showed that this effect was driven primarily by greater attenuation of the P3a in response to familiar alcohol cues in hazardous drinkers (Figure 4; b = –0.179, SE = 0.06, *p* = 0.003). To test whether the P3b component related to cue-specific POMDP parameters between groups, we ran two models with group, cue type, and P3b amplitude as predictors for BONUS in one model and IEV in the other. IEV coefficients were predicted by a three-way interaction between group, chosen cue type, and P3b amplitude (b = 0.198, SE = 0.097, *p* = 0.045). P3b amplitude was associated with increased IEV of alcohol cues for hazardous drinkers rather than for controls (Figure 5; b = 0.171, SE = 0.074, *p* = 0.02). In sum, hazardous drinkers were specifically characterized by a P3b marker of alcohol-relevant exploitation and a P3a marker of alcohol-relevant exploration, identifying discrete neural systems underlying alcohol-related decision-making.

## Discussion

The present study is the first to show that hazardous drinkers demonstrate an alcohol cue-specific bias in the explore-exploit tradeoff. Hazardous drinkers explore alcohol cues more often than control participants and prefer to exploit familiar options when presented with novel non-alcohol cues. A preference for exploring novel alcohol cues in hazardous drinkers was also tied to the P3a. Critically, POMDP-derived value estimates provided a mechanistic bridge for interpreting the latent neuronal computations linking the observed P3 amplitudes and choice behaviors. The relative valuation of novel cues (i.e., exploration BONUS) was associated with enhanced P3a amplitudes *and* exploratory choice in hazardous drinkers, suggesting that brain networks encoding this motivational signal (Hogeveen et al., 2022) may be preferentially biased in hazardous drinkers. Conversely, P3b amplitudes were not sensitive to the value of exploration in hazardous drinkers but were associated with an increased sensitivity to the IEV of exploiting familiar alcohol imagery. Given the known functional roles of the P3a and P3b components (Polich, 2007; Cavanagh, 2019; Donchin & Coles, 1988; Rac-Lubashevsky & Kessler, 2019; Twomey et al., 2015), these EEG responses to novel and familiar alcohol cues may act as sensitive markers of alcohol-specific novelty-induced decision-making and information updating in hazardous drinkers, respectively.

In addition to enhanced P3b associations with the IEV of alcohol cues, hazardous drinkers also preferentially exploited familiar alternatives when presented with novel non-alcohol cues. These findings utilizing alcohol versus non-alcohol cues complicate prior research on addiction and the explore-exploit tradeoff which has typically indicated generalized reductions in adaptive exploratory decision-making in response to neutral stimuli (Addicott et al., 2017; Cogliati Dezza et al., 2018). For example, recent work on methamphetamine use disorder found an elevated exploration BONUS is related to reductions in methamphetamine use (Robinson et al., 2022). However, existing studies have not leveraged drug-specific stimuli. In light of the alcohol cue-specific nature of the current findings, there is additional complexity of interpreting computational neuroscience findings when restricted to neutral cues. Explore-exploit decision-making in response to drug-specific cues could therefore represent an understudied component of the formation and maintenance of addictive behaviors. The degree to which dissociable decision-making processes in the face of addiction-relevant imagery is observed at different stages of addiction, or within subtypes of hazardous drinkers, may have the potential to inform precision medicine approaches to AUD treatment.

The P3a is associated with processing novel stimuli and is thought to relate to dopaminergic frontocortical activation (Polich, 2007; Huang et al., 2015; Bhakta et al., 2022). Present findings demonstrated that hazardous drinkers showed differential P3a amplitudes dependent on their selection of the novel or best alternative stimulus—P3a amplitude was higher when exploring alcohol cues and lower when choosing to exploit familiar alternatives in lieu of novel non-alcohol cues. This suggests the P3a novelty orienting potential may preferentially bias action selection depending on the motivational salience of the novel cue. The POMDP results help to clarify the algorithms through which novel alcohol cues may acquire increased motivational salience relative to novel non-alcohol cues in hazardous drinkers. Specifically, P3a novelty orienting responses to alcohol cues were correlated with enhanced weighting of the relative value of exploration in hazardous drinkers. Consistent with the P3a link to dopamine, enhancing extracellular dopamine increases the selection of novel choice options in a variant of the current task (Costa et al., 2014; Djamshidian et al., 2011) and is associated with activation of the dopaminergic midbrain (Wittmann et al., 2008). Encoding of these relative future value computations during exploration has been observed across frontopolar, frontoparietal, and mesocorticolimbic circuitry in humans and nonhuman primates (Costa et al., 2019; Hogeveen et al., 2022; Tang et al., 2022). Multimodal EEG-fMRI studies will be critical to exploring whether trial-to-trial variance in P3a novelty orienting responses at frontal electrodes are modulated by the encoding of exploration BONUS signals across distributed brain networks.

We also observed a modest association between P3b responses and the tendency to exploit high IEV alcohol cues in hazardous drinkers. The P3b is observed in posterior cortex and is thought to reflect mnemonic and information updating processes (Polich, 2007; Donchin & Coles, 1988; Rac-Lubashevsky & Kessler, 2019; Twomey et al., 2015). Therefore, whereas the frontal P3a response likely represents a marker of enhanced novelty-orienting and alcohol-specific exploration, the more posterior P3b component may represent a marker of enhanced updating/maintenance of the immediate value of familiar alcohol cues among hazardous drinkers.

The present study was primarily limited in that hazardous drinking participants could not be categorized as having AUD at the time of the study. Although 24 of the 26 hazardous drinking participants had been diagnosed with AUD either at the time of enrollment in ABQDrinQ and/or within their lifetime, future research should implement this task with larger samples of individuals who meet diagnostic criteria for AUD during testing. Additionally, non-appetitive neutral control images rather than beverages could be added to future iterations of the task to characterize the behavioral and neural processes driving alcohol-specific exploration more precisely.

In summary, hazardous drinkers showed enhanced exploration of alcohol cues and enhanced exploitation of familiar alternatives to non-alcohol cues. These behaviors were tied to changes in the novelty-sensitive P3a. Hazardous drinkers also showed enhanced relative valuation of novel alcohol options across trials via the exploration BONUS which was also linked to the P3a. Altogether, these results suggest dissociable neural signals coding the information that informs potentially maladaptive decision-making processes in hazardous alcohol use. Future studies should leverage substance-specific stimuli in the explore-exploit paradigm to further illuminate how distinct behaviors in substance use and addiction are potentially driven by specific neurocomputational processes.

## Additional File

The additional file for this article can be found as follows:

10.5334/cpsy.96.s1 Supplemental File. Supplemental Table 1 and Figure 1.
