# Supplementary material for: Electrophysiological Markers of Aberrant Cue-Specific Exploration in Hazardous Drinkers
Source: Comput Psychiatr. 2023 Jul 28;7(1):47–59. doi: 10.5334/cpsy.96 (PMC11104413; doi:10.5334/cpsy.96)
Supplement: Supplemental File. — Supplemental Table 1 and Figure 1. [file cpsy-7-1-96-s1.pdf]

Supplemental Table 1

Group Demographics

		Controls	Hazardous Drinkers
N (female)		26 (15)	27 (15)
Age (SD)		37.2 (10.7)	38.5 (9.59)
Years of education (SD)		16.1 (3.02)	15 (2.34)
AUDIT (SD)		1.31 (0.928)	10.6 (5.64)
BDI (SD)		4.62 (7.84)	11.4 (12.2)
Ethnicity	Hispanic:	9	9
	Non-Hispanic:	17	18
Race	Asian:	3	0
	African American:	0	0
	Caucasian:	18	17
	Native American/Alaskan:	2	5
	Native Hawaiian/Pacific Islander:	0	0
	Mixed race:	0	1
	Other:	1	3

Supplemental Figure 1

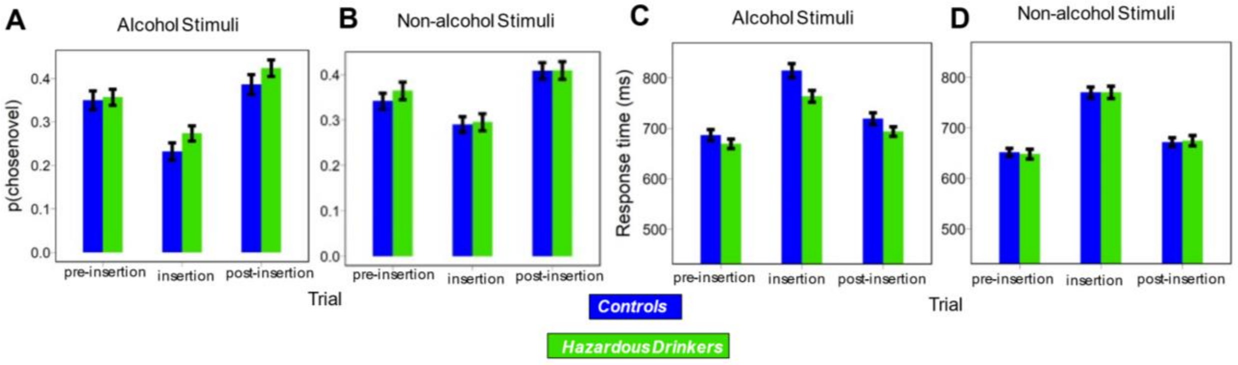

(A) Probability of selecting the novel stimulus for alcohol (A) and non-alcohol (B) stimuli between groups around novel insertion trials. Response time in milliseconds between groups for alcohol (C) stimuli and non-alcohol (D) stimuli around novel insertion trials.
